# Inexperienced clinicians can extract pathoanatomic information from MRI narrative reports with high reproducibility for use in research/quality assurance

**DOI:** 10.1186/2045-709X-19-16

**Published:** 2011-07-21

**Authors:** Peter Kent, Andrew M Briggs, Hanne B Albert, Andreas Byrhagen, Christian Hansen, Karina Kjaergaard, Tue S Jensen

**Affiliations:** 1 Research Department, Spine Centre of Southern Denmark, Lillebaelt Hospital, Institute of Regional Health Services Research, University of Southern Denmark, Middelfart, Denmark; 2 School of Physiotherapy and Curtin Health Innovation Research Institute, Curtin University, Perth, Australia; 3 Odense Physiotherapy Sports and Fitness, Odense, Denmark; 4 Quick Care, Odense, Denmark; 5 Southwest Jutland Hospital, Esbjerg, Denmark

**Keywords:** MRI, narrative report, coding, spine, pathoanatomy

## Abstract

**Background:**

Although reproducibility in reading MRI images amongst radiologists and clinicians has been studied previously, no studies have examined the reproducibility of inexperienced clinicians in extracting pathoanatomic information from magnetic resonance imaging (MRI) narrative reports and transforming that information into quantitative data. However, this process is frequently required in research and quality assurance contexts. The purpose of this study was to examine inter-rater reproducibility (agreement and reliability) among an inexperienced group of clinicians in extracting spinal pathoanatomic information from radiologist-generated MRI narrative reports.

**Methods:**

Twenty MRI narrative reports were randomly extracted from an institutional database. A group of three physiotherapy students independently reviewed the reports and coded the presence of 14 common pathoanatomic findings using a categorical electronic coding matrix. Decision rules were developed after initial coding in an effort to resolve ambiguities in narrative reports. This process was repeated a further three times using separate samples of 20 MRI reports until no further ambiguities were identified (total n = 80). Reproducibility between trainee clinicians and two highly trained raters was examined in an arbitrary coding round, with agreement measured using percentage agreement and reliability measured using unweighted Kappa (*k*). Reproducibility was then examined in another group of three trainee clinicians who had not participated in the production of the decision rules, using another sample of 20 MRI reports.

**Results:**

The mean percentage agreement for paired comparisons between the initial trainee clinicians improved over the four coding rounds (97.9-99.4%), although the greatest improvement was observed after the first introduction of coding rules. High inter-rater reproducibility was observed between trainee clinicians across 14 pathoanatomic categories over the four coding rounds (agreement range: 80.8-100%; reliability range *k *= 0.63-1.00). Concurrent validity was high in paired comparisons between trainee clinicians and highly trained raters (agreement 97.8-98.1%, reliability *k *= 0.83-0.91). Reproducibility was also high in the second sample of trainee clinicians (inter-rater agreement 96.7-100.0% and reliability *k *= 0.76-1.00; intra-rater agreement 94.3-100.0% and reliability *k *= 0.61-1.00).

**Conclusions:**

A high level of radiological training is not required in order to transform MRI-derived pathoanatomic information from a narrative format to a quantitative format with high reproducibility for research or quality assurance purposes.

## Background

Magnetic resonance imaging (MRI) is considered the gold standard modality for imaging spinal structures *in vivo *[1]. Spinal pathoanatomy may be readily visualised using MRI and some pathoanatomic features, such as tumours, fractures, infections and nerve root compression, are usually clinically important. On the other hand, the clinical relevance of some other spinal pathoanatomic features, particularly their association with spinal pain and other symptoms, remains uncertain and is an ongoing topic of debate [2-4]. Although further exploration of this issue may be warranted, particularly in the context of vertebral endplate signal changes, which have shown an association with pain [5], a prerequisite for such investigations is the reliable extraction of information from MRI images.

Quantitative coding of MRI findings is important where such data are used for research and quality assurance purposes. For example, narrative MRI reports need to be routinely transformed into quantitative data within the context of clinical trials, cohort studies and health registries. Although such quantification can be performed directly by experienced radiologists who have been trained in the relevant research protocols, this is often not practical because there is a widespread shortage of experienced radiologists, especially research radiologists, and their participation is usually expensive. Therefore, an alternative pathway is for MRI findings to be quantified from narrative reports dictated by radiologists.

There are two important interpretative steps in the pathway from acquiring spinal MR images to a quantification of any identified pathoanatomic findings. Reproducibility (agreement and reliability) in each step is important for best practice research. First, radiologists need to interpret the scan images and dictate a narrative report that adequately describes the relevant normal and abnormal pathoanatomic findings. Several studies have described the inter-radiologist reliability of this process as typically being fair to substantial (Kappa's ranging from 0.21 to 0.80) [1,6-8]. Second, researchers who review narrative reports need to extract important pathoanatomic findings in a consistent manner. Although earlier research has identified comparable reproducibility between radiologists and spinal surgeons, neurosurgeons and conservative-care clinicians in reading spinal MR images [1], the reproducibility of researchers in extracting information from MRI narrative reports and transforming this information into a quantitative format has not been evaluated previously.

It remains uncertain as to what level of clinical training is required to extract pertinent pathoanatomic information from narrative reports with adequate reproducibility for research/quality assurance purposes. Therefore, the aim of this study was to quantify the reproducibility of an inexperienced group of trainee clinicians in extracting pathoanatomic information from MRI narrative reports.

## Methods

### Design

A repeated measures study was performed to investigate inter-rater reproducibility in transforming pathoanatomic information from narrative MRI reports into quantitative data. Two groups of three final year (third year) physiotherapy students who had received minimal training in interpreting MRI narrative reports were engaged as independent raters. They had only received two lectures introducing them to the broad array of imaging techniques. The study was covered by a quality assurance approval by the Scientific Ethics Committee of Southern Denmark and complied with the Declaration of Helsinki (2008).

Conceptually, reproducibility contains two components - agreement and reliability [9]. Agreement is about measurement error and quantifies the similarity of scores obtained by repeated measurement. This is a useful measure when the measurement tool and measurement process being examined are to be used for *monitoring change over time*. Reliability is about how well the measurement tool and measurement process are able to *distinguish between subjects or objects *(for example people or pathologies), despite the measurement error. Reliability is influenced by the variability in the scores. Therefore, reliability may vary within a study population (for example from one body region to another) or between study populations, even though the measurement error remains the same [9].

### Imaging

In the MRI unit of the Spine Centre of Southern Denmark, there were narrative reports of lumbar MRIs available for a total of 4,233 community-dwelling individuals who had attended the Centre over the previous 8 years. MRIs of other body regions or repeat lumbar MRIs for individuals were not included in that sample. This spine centre is a publicly-funded secondary care outpatient facility specialising in the diagnosis and management of spinal pain, and is located on the island of Funen, Denmark. Patients were referred to the Centre by primary care chiropractors and general practitioners between the years 2000 and 2008.

A standard lumbar MRI protocol utilising a 0.2 T MRI system (Magnetom Open Viva, Siemens AG, Erlangen, Germany) was used. All patients were placed in the supine position with straightened legs. The imaging protocol consisted of one localiser and four imaging sequences - T1 and T2 sagittal and axial images (more detail is available on request from the authors).

Axial images were performed on the three lower lumbar levels. If serious pathology were present or herniations were located at higher lumbar levels, relevant supplementary sequences were performed. All images over this eight-year period were read by either one of two experienced musculoskeletal radiologists.

### Coding of MRI narrative reports

**Figure 1:**
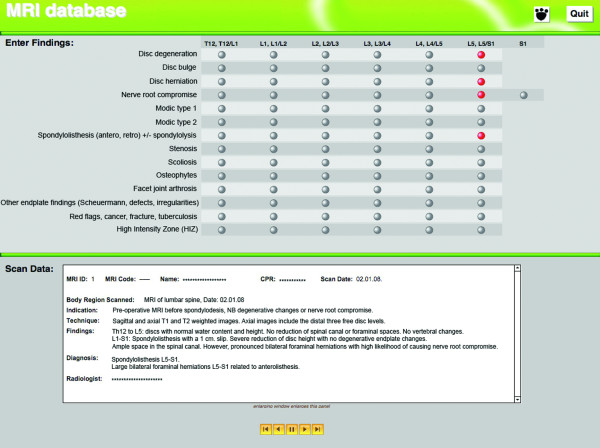
**Screen shot of electronic coding matrix, consisting of 14 pathoanatomic categories for coding a narrative MRI report**.

An electronic coding matrix was developed for this study using FileMaker Pro 9 (FileMaker Inc, CA, USA). The coding matrix was designed to facilitate data capture for subsequent research projects. This coding matrix was used by the three raters to transform findings from narrative reports into a quantitative measure (yes/no score) of the presence of 14 possible pathologies including: intervertebral disc degeneration, disc bulge, disc herniation, nerve root compromise, Modic change type 1, Modic change type 2, spondylolisthesis (anterior or retro) with or without spondylolysis, stenosis, scoliosis, osteophytes, facet joint arthrosis, other endplate irregularities (including Scheuermann defects, irregularities), red flags (tumour, fracture, infection), and high intensity zones (Figure 1). These pathologies were chosen as they were pertinent to research undertaken in the study context. Other pathologies may have been reported in the narrative reports, but as they were not relevant to our research, they were not coded by the raters. The raters used the matrix to indicate whether pathoanatomic features were reported as present between vertebral levels T12-L5, including an option to nominate compromise of the S1 nerve root. For the purpose of coding, a vertebral segment was defined as extending from the superior vertebral endplate to the caudal aspect of the intervertebral space below, for example the T12 vertebra and T12/L1 inter-vertebral space.

An initial sample of 20 reports (0.47%) was randomly selected from all the people in the database who shared the same weekday of their date of birth. All coding was performed independently by the first group of three raters who remained blinded to each other's scores throughout the study. As each rater coded each MRI, whenever a narrative report finding was considered ambiguous, the rater(s) noted that ambiguity on a log sheet and selected the pathoanatomic feature in the coding matrix that they believed to be the most appropriate. At the completion of this initial cycle of coding 20 reports (a coding round), ambiguous report findings were collated and decision rules developed by senior researchers to provide a consistent decision-making process between raters. This group of inexperienced raters was able to contribute to the formation of the coding rules and provide feedback on their utility.

The initial coding round was undertaken without any decision rules. Another separate sample of 20 MRI reports of people who shared a different birth day was then selected and this process was repeated until no further ambiguities in reporting were identified by the raters. Three iterations of the coding rules were developed, therefore the total number of MRIs used was 80 (1.9% of the total available sample). The final set of decision rules is included as Additional file 1. In the first round of coding, three categories were not included in the matrix ('other endplate irregularities', 'red flags', and 'high intensity zones') but were added in the second round to refine the specificity of findings.

The choice of 20 MRI reports per round was arbitrary as we had no data *a priori *on which to estimate the likely reliability between raters and its variance, nor the likely proportions of positive diagnoses. Therefore, we performed a *post hoc *power calculation to determine the reliability that was statistically detectable, given the sample size, Kappa values, and observed proportion of positive diagnoses, using the method described by Sim and Wright [10].

### Inter-rater reproducibility

Inter-rater reproducibility was calculated separately for coding rounds 1 to 4. Given the uncertainty as to what level of clinical training was required to achieve adequate reproducibility in extracting pathoanatomic information from the narrative reports, we compared the reproducibility of the three trainee clinicians to that of two highly trained raters during an arbitrary coding round (round two). The two highly trained raters were experienced researchers in the field of spinal MRI, including designing and conducting reproducibility studies of lumbar MRI findings [11,12]. One was a chiropractor and the other a physiotherapist, both of whose PhDs and post-doctoral experience focused on the investigation of MRI findings.

Inter-rater reproducibility was also calculated for the coding round that involved the second group of three trainee clinicians. These raters were exposed to the coding rules but did not participate in rule formation. Their training consisted solely of receiving feedback on their reproducibility when coding five randomly selected MRI reports *not used *in the coding rounds in this study. Therefore, their performance was more representative of the reproducibility that might be achievable if these coding rules were used in other clinical or research settings.

### Intra-rater reproducibility (test-retest)

The second group of trainee clinicians also recoded the same 20 MRI reports one week after their initial coding round. They were blinded to their initial coding scores. These two sets of coding results were used to calculate intra-rater reproducibility.

### Data Analysis

The raw coding data were exported from the Filemaker Pro 9 database into Microsoft Excel 2008 (Microsoft Corp, Redmond, WA, USA). This allowed the coding matrices for each MRI report to be aligned according to rater.

Inter-rater and intra-rater agreement were quantified using percentage agreement. Percentage agreement expresses the proportion of cases where there was agreement between the two raters' responses, that is, where both raters nominated the presence of a particular condition or concurred on the absence of a that condition. It was calculated with a method reported in other reliability studies of MRI findings [12-14] using Microsoft Excel 2008. This method involved: (a) summing the number of agreements in each paired comparison, (b) expressing that sum as a proportion (percentage) of the number of ratings, (c) repeating this for all possible pairs of raters in that coding round, and (d) calculating the mean and 95% confidence interval of those proportions to provide a single estimate of the percentage agreement for that coding round.

Inter-rater and intra-rater reliability was quantified using the unweighted Kappa statistic. The Kappa coefficient expresses reliability for nominal or ordinal ratings while accounting for chance agreement [15]. The magnitude of Kappa can be influenced by prevalence of the condition, bias and non-independence of ratings [10] Kappa coefficients were calculated using the 'kap' and 'kapci' procedures in STATA 10.1 (Stata Corp, College Station, Texas, USA). We used a STATA program (do-file) that automated the process of: (i) calculating Kappa for all the possible paired comparisons, (ii) averaging Kappa values by first transforming them into Z-scores (Fisher's transformation), (iii) finding the arithmetic mean and calculating the 95% confidence interval, and then (iv) transforming the results back into Kappa values. Consistent with recommendations in the literature [6,16,17], the program did not calculate Kappa when the prevalence of a pathoanatomic finding was less than 10% or greater than 90%, as in situations of very high or very low prevalence, the chance agreement increases and biases the value of Kappa [18]. In the current study, this restriction applied to the prevalence of the pathology at each and every vertebral level being compared, and for findings of all raters being compared.

Agreement (percentage agreement) and reliability (Kappa) were also calculated between the highly trained raters and between the highly trained raters and trainee clinicians. Comparisons between highly trained raters and trainee clinicians represent a measure of concurrent validity.

Percentage agreement and Kappa were calculated in two ways: (1) using only the ratings for each separate pathology to allow comparisons of reproducibility between pathologies, and (2) using all the ratings across all pathologies to give a global summary statistic. For the calculation of percentage agreement and Kappa for each pathology, there were between 120 (one pathology × 6 vertebral levels × 20 MRI reports) and 140 ratings (only for nerve root irritation, one pathology × 7 vertebral levels × 20 MRI reports) in each pair-wise comparison between inexperienced raters in each coding round. This method was used when determining the inter-rater or intra-rater reproducibility of either group of trainee clinicians.

To calculate a global summary statistic for either percentage agreement or Kappa, all the ratings for a coding round were used in a single comparison. This was a total of 1700 potential ratings in each pair-wise cross-pathology comparison between raters for each round ((12 pathologies × 6 vertebral levels + nerve root irritation × 7 vertebral levels) × 20 MRI reports = 1700). This method was used when determining the reproducibility between the highly trained raters and also between the highly trained raters and trainee clinicians, as although this summary statistic provides less detail, it is easier to interpret.

The prevalence for each pathology was calculated for each coding round. This was performed on a 'whole person' level, that is, where the presence of a particular pathology in a person at one or more vertebral levels was counted as a single instance.

## Results

The average age of the people whose MRIs were included was 51.4 years (SD 13.1; full range 27 to 88), and 55.0% were female. The prevalence of the 14 pathoanatomic categories within each sample of 20 MRI scans ranged from 0.0% to 100.0% (Tables 1 and 2). All pathologies were present in at least one of the five coding rounds.

**Table 1:** Inter-rater reproducability results for the initial group of three trainee clinicians for 14 pathoanatomic categories across the four coding rounds, expressed as a Kappa co-efficient and percentage agreement with 95% confidence intervals (95% CI)

Pathoanatomic category	Reliability index (95%CI)	Coding round 1	Coding round 2	Coding round 3	Coding round 4
Intervertebral disc degeneration	Prevalence* Kappa Percentage agreement	71.7% (50.2-93.1) *0.66 (0.54-0.77)* 80.8% (73.8-87.9)	94.0 (92.0-96.0) *0.94 (0.84-1.00)* 95.8 (92.3-99.4)	80.0 (80.0-80.0) *n/a* 98.2 (95.8-100.0)	83.3 (80.1-86.6) *0.91 (0.80-1.00)* 97.5 (94.7-100.0)
	
Intervertebral disc bulge	Prevalence *Kappa* Percentage agreement	73.3% (70.1-76.6) *0.91 (0.78-1.00)* 95.0% (94.7-100.0)	87.0 (83.1-90.9) *0.86 (0.76-0.90)* 92.5 (87.7-97.2)	70.0 (70.0-70.0) *1.00 (0.87-1.00)* 100.0	48.3 (45.1-51.6) *0.89 (0.76-1.00)* 99.2 (97.5-100.0)
	
Intervertebral disc herniation	Prevalence *Kappa* Percentage agreement	40.0% (34.3-45.7) *0.91 (0.73-1.00)* 97.5% (94.7-100.0)	61.0 (59.0-63.0) *1.00 (0.85-1.00)* 100.0	40.0 (40.0-40.0) *0.98 (0.87-1.00)* 96.5 (93.1-99.9)	45.0 (45.0-45.0) *1.00 (0.82-1.00)* 100.0
	
Nerve root compromise	Prevalence *Kappa* Percentage agreement	13.3% (4.7-22.0) *n/a* 97.5% (94.7-100.0)	42.0 (31.9-52.1) *0.74 (0.49-1.00)* 94.2 (90.0-98.4)	31.7 (23.0-40.3) *0.63 (0.45-0.81)* 93.0 (88.3-97.7)	18.3 (15.1-21.6) 1*.00 (0.75-1.00)* 99.2 (97.5-100.0)
	
Modic change type 1	Prevalence *Kappa* Percentage agreement	26.7% (23.4-29.9) *0.86 (0.68-1.00)* 95.0% (91.1-98.1)	18.0 (15.6-20.4) *0.86 (0.60-1.00)* 98.3 (96.0-100.0)	20.0 (20.0-20.0) *1.00 (0.82-1.00)* 100.0	18.3 (15.1-21.6) *0.86 (0.60-1.00)* 100.0
	
Modic change type 2	Prevalence *Kappa* Percentage agreement	25.0% (19.3-30.7) *0.76 (0.51-1.00)* 97.5% (94.7-100.0)	10.0 (5.6-14.4) *n/a* 98.3 (96.0-100.0)	20.0 (20.0-20.0) *1.00 (0.82-1.00)* 100.0	5.0 (5.0-5.0) *n/a* 100.0
	
Spondylolisthesis	Prevalence *Kappa* Percentage agreement	20.0% (20.0-20.0) *n/a* 97.5% (94.7-100.0)	19.0 (17.0-21.0) *1.00 (0.75-1.00)* 100.0	10.0 (10.0-10.0) *1.00 (0.82-1.00)* 100.0	0.0 (0.0-0.0) *n/a* 100.0
	
Stenosis	Prevalence *Kappa* Percentage agreement	6.7% (3.4-9.9) *n/a* 97.5% (94.7-100.0)	23.0 (20.6-25.4) *0.84 (0.59-1.00)* 99.2 (97.5-100.0)	25.0 (25.0-25.0) *0.95 (0.74-1.00)* 99.1 (97.4-100.0)	0.0 (0.0-0.0) *n/a* 100.0
	
Scoliosis	Prevalence *Kappa* Percentage agreement	25.0% (25.0-25.0) *1.00 (0.82-1.00)* 100.0%	6.0 (4.0-8.0) *n/a* 95.0 (91.1-98.9)	5.0 (5.0-5.0) *n/a* 99.1(97.4-100.0)	5.0 (5.0-5.0) *n/a* 100.0
	
Osteophytes	Prevalence *Kappa* Percentage agreement	5.0% (5.0-5.0) *n/a* 100.0%	1.0 (0.0-3.0) *n/a* 100.0	10.0 (10.0-10.0) *n/a* 99.1 (97.4-100.0)	10.0 (10.0-10.0) *1.00 (0.75-1.00)* 99.2 (97.5-100.0)
	
Facet joint arthrosis	Prevalence *Kappa* Percentage agreement	0.0% (0.0) *n/a* 100.0%	14.0 (12.0-16.0) *0.68 (0.42-0.93)* 95.0 (91.1-98.9)	20.0 (20.0-20.0) *1.00 (0.82-1.00)* 100.0	5.0 (5.0-5.0) *n/a* 100.0
	
Endplate irregularities	Prevalence *Kappa* Percentage agreement	^#^	1.0 (0.0-3.0) *n/a* 99.2 (97.5-100.0)	25.0 (19.3-30.7) *1.00 (0.82-1.00)* 98.2 (95.8-100.0)	18.3 (11.8-24.9) *0.74 (0.56-0.92)* 97.5 (94.7-100.0)
	
Red flags	Prevalence (%) *Kappa* Percentage agreement	^#^	0.0 (0.0-0.0) n/a 100.0	8.3 (1.8-14.9) *n/a* 98.2 (95.8-100.0)	0.0 (0.0-0.0) *n/a* 100.0
	
High intensity zone	Prevalence (%) *Kappa* Percentage agreement	^# ^	15.0 (11.9-18.1) *0.84 (0.59-1.00)* 99.2 (97.5-100.0)	26.7 (20.1-33.2) *0.85 (0.67-1.00)* 98.2 (95.8-100.0)	25.0 (25.0-25.0) *1.00 (0.82-1.00)* 100.0

* The prevalence estimates are calculated on a 'whole person' level. That is, the presence of a particular pathology in a person at one or more vertebral levels was counted as a single instance.

95%CI = 95% confidence interval in brackets.

^# ^not included in coding round 1.

n/a = kappa co-efficient not calculated. Calculation of kappa in STATA requires the prevalence of the pathology to be 10% to 90% at each and every vertebral level being compared. This restriction applies to the findings of all raters being compared.

**Table 2:** Inter-rater reproducability between trainee clinicians and highly trained raters within a single coding round, expressed as a paired-comparison percentage agreement and Kappa co-efficient with 95% confidence intervals (95% CI)

Paired comparison	Percentage agreement (95% CI)	Kappa (95% CI, p value)
Highly trained rater 1 × Trainee clinician 1	98.2% (97.5%-98.8%)	0.87 (0.82-0.91)
Highly trained rater 1 × Trainee clinician 2	97.8% (97.2%-98.5%)	0.85 (0.79-0.89)
Highly trained rater 1 × Trainee clinician 3	97.5% (96.8%-98.2%)	0.83 (0.77-0.87)
*Mean*	*97.8% (97.0%-98.7%)*	

Highly trained rater 2 × Trainee clinician 1	98.1% (97.4%-98.7%)	0.87 (0.82-0.90)
Highly trained rater 2 × Trainee clinician 2	97.5% (96.8%-98.2%)	0.83 (0.77-0.87)
Highly trained rater 2 × Trainee clinician 3	98.6% (98.1%-99.2%)	0.91 (0.86-0.94)
*Mean*	*98.1% (96.7%-99.5%)*	

### Reproducibility during the formation of the coding rules

Overall, the mean paired-comparison percentage agreement between the initial three trainee clinicians across the 14 pathoanatomic categories increased with each subsequent round of coding from 97.9% to 99.4%. Overall, across the 14 categories and four rounds of coding, their inter-rater agreement (percentage agreement) ranged from 80.8%-100%, and reliability (Kappa) ranged from 0.63-1.00 (Table 1). The inter-rater reproducibility between highly trained raters determined in a single coding round, expressed as percentage agreement (95% CI) was 97.3% (96.6-98.1%) and Kappa (95% CI) was 0.82 (0.77-0.86). Kappa coefficients greater than 0.80 are generally considered to represent excellent reliability [19].

For the round in which the initial trainee clinicians were compared with highly trained raters, the mean percentage agreement between the trainee clinicians and highly trained rater 1 was 97.8% (97.0%-98.7%) and with highly trained rater 2 was 98.1% (96.7%-99.5%), suggesting excellent agreement. The Kappa coefficients ranged from 0.83-0.87 for comparisons between these trainee clinicians and highly trained rater 1, and 0.83-0.91 when compared with highly trained rater 2, (Table 2).

### Reproducibility in inexperienced clinicians trained with the final set of coding rules

Overall, across the 14 categories and single round of coding, the second group of inexperienced clinicians displayed an *inter-rater *agreement (percentage agreement) ranging from 96.7-100.0% and their reliability (Kappa) ranged from 0.76-1.00 (Table 3). Their test-retest reproducibility was also high, with *intra-rater *agreement ranging from 94.3-100.0% and reliability ranging from *k *= 0.61-1.00.

**Table 3:** Inter-rater reproducability reliability results for the second group of three trainee clinicians for 14 pathoanatomic categories across a single coding round, expressed as a Kappa co-efficient and percentage agreement with 95% confidence intervals (95% CI)

Pathoanatomic category	Reliability index (95%CI)	Inter-rater Coding round 1	Inter-rater Coding round 2	Test-retest (intra-rater)
Intervertebral disc degeneration	Prevalence* Kappa Percentage agreement	90.0% (90.0-90.0) *0.99 (0.87-1.00)* 99.3% (99.0-100.0)	90.0% (90.0-90.0) *0.93 (0.82-1.00)* 96.7% (95.0-99.0)	- *0.88 to 1.00* 97.3% (94.0-100.0)
	
Intervertebral disc bulge	Prevalence *Kappa* Percentage agreement	90.0% (90.0-90.0) *0.96 (0.84-1.00)* 98.0% (97.0-99.0)	85.0% (79.3-90.7) *0.93 (0.80-1.00)* 96.7% (95.0-99.0)	- *0.79 to 0.96* 94.3% (91.0-97.0)
	
Intervertebral disc herniation	Prevalence *Kappa* Percentage agreement	58.3% (55.1-61.6) *0.94 (0.80-1.00)* 98.7% (98.0-99.0)	58.3% (55.1-61.6) *0.94 (0.80-1.00)* 98.7% (98.0-100.0)	- *0.86 to 0.91* 97.3% (97.0-98.0)
	
Nerve root compromise	Prevalence *Kappa* Percentage agreement	53.3% (50.1-56.6) *0.76 (0.58-0.94)* 96.0% (94.0-98.0)	55.0% (49.3-60.7) *0.81 (0.63-0.99)* 97.3% (96.0-98.0)	- *0.61 to 0.86* 95.3% (94.0-98.0)
	
Modic change type 1	Prevalence *Kappa* Percentage agreement	20.0% (20.0-20.0) *1.00 (0.82-1.00)* 100.0% (100.0-100.0)	21.7% (18.4-24.9) *0.93 (0.75-1.00)* 99.3% (99.0-100.0)	- *0.89 to 1.00* 99.7% (99.0-100.0)
	
Modic change type 2	Prevalence *Kappa* Percentage agreement	10.0% (10.0-10.0) *1.00 (0.75-1.00)* 100.0% (100.0-100.0)	8.3% (5.1-11.6) *n/a* 99.3% (99.0-100.0)	- *1.00 to 1.00* 99.7% (99.0-100.0)
	
Spondylolisthesis	Prevalence *Kappa* Percentage agreement	20.0% (20.0-20.0) *1.00 (0.75-1.00)* 100.0% (100.0-100.0)	20.0% (20.0-20.0) *1.00 (0.75-1.00)* 100.0% (100.0-100.0)	- *1.00 to 1.00* 100.0% (100.0-100.0)
	
Stenosis	Prevalence *Kappa* Percentage agreement	16.7% (13.2-19.9) *n/a* 99.3% (99.0-100.0)	15.0% (15.0-15.0) *n/a* 100.0% (100.0-100.0)	- *n/a* 99.7% (99.0-100.0)
	
Scoliosis	Prevalence *Kappa* Percentage agreement	5.0% (5.0-5.0) *n/a* 100.0% (100.0-100.0)	5.0% (5.0-5.0) *n/a* 100.0% (100.0-100.0)	- *n/a* 100.0% (100.0-100.0)
	
Osteophytes	Prevalence *Kappa* Percentage agreement	0.0% (0.0-0.0) *n/a* 100.0% (100.0-100.0)	0.0% (0.0-0.0) *n/a* 100.0% (100.0-100.0)	- *n/a* 100.0% (100.0-100.0)
	
Facet joint arthrosis	Prevalence *Kappa* Percentage agreement	15.0% (15.0-15.0) *1.00 (0.82-1.00)* 100.0% (100.0-100.0)	15.0% (15.0-15.0) *0.92 (0.74-1.00)* 98.7% (98.0-100.0)	- *0.88 to 1.00* 99.3% (98.0-100.0)
	
Endplate irregularities	Prevalence *Kappa* Percentage agreement	5.0% (5.0-5.0) *n/a* 100.0% (100.0-100.0)	1.7% (0.0-4.9) *n/a* 99.3% (99.0-100.0)	- *n/a* 99.3% (99.0-100.0)
	
Red flags	Prevalence *Kappa* Percentage agreement	0.0% (0.0-0.0) *n/a* 100.0% (100.0-100.0)	0.0% (0.0-0.0) *n/a* 100.0% (100.0-100.0)	- *n/a* 100.0% (100.0-100.0)
	
High intensity zone	Prevalence *Kappa* Percentage agreement (%)	15.0% (15.0-15.0) *1.00 (0.75-1.00)* 100.0% (100.0-100.0)	15.0% (15.0-15.0) *1.00 (0.75-1.00)* 100.0% (100.0-100.0)	- *n/a* 100.0% (100.0-100.0)

* The prevalence estimates are calculated on a 'whole person' level. That is, the presence of a particular pathology in a person at one or more vertebral levels was counted as a single instance.

95%CI = 95% confidence interval in brackets.

n/a = kappa co-efficient not calculated. Calculation of kappa in STATA requires the prevalence of the pathology to be 10% to 90% at each and every vertebral level being compared. This restriction applies to the findings of all raters being compared.

### Sample size

Post-hoc observations showed that across all patient MRI reports, all pathologies and all vertebral levels, the rater endorsement of the presence of a pathology was approximately 8%. A post-hoc power calculation showed that on the basis of an observed proportion of positive diagnoses (rater endorsement) of 10%, and an assumed null hypothesis value of Kappa to be 0.40, then a sample size of 102 ratings would detect with 80% power, a statistically significant Kappa of 0.90. As the sample sizes used in this study for the calculation of Kappa usually ranged between 120 and 140 observations (within pathology comparisons), and for some calculations was as large as 1700 observations (across all pathology comparisons), there was adequate power in the sample.

## Discussion

This study is the first to establish that inter-rater and intra-rater reproducibility in extracting pathoanatomic information from MRI narrative reports is excellent. Moreover, we have demonstrated that a high level of radiological experience is not required to perform this task well, even in the absence of coding rules. These findings have useful implications for conducting large-scale research and quality assurance projects that include MRI data.

Agreement between the initial group of trainee clinicians exceeded a percentage agreement of 90%, except for intervertebral disc degeneration in coding round 1 (80.8%). The greater discordance between raters for intervertebral disc degeneration may be explained by the larger number of terms used by radiologists to characterise degeneration, relative to other pathoanatomic findings. Nonetheless, with the introduction of coding rules, the percentage agreement for this category increased to a maximum of 98.2% and was comparable to that of highly trained raters (97.3%). The greatest improvement in inter-rater agreement for intervertebral disc degeneration was observed after the first introduction of coding rules (round 2). This observation is consistent with the improvement in the mean percentage agreement across the 14 pathoanatomic categories also being largest between coding rounds 1 and 2 (0.8%). Although the mean percentage agreement continued to improve with the two subsequent iterations of the coding rules, the improvements were relatively minor (0.3-0.4%), indicating only a small additional training effect. This suggests that the initial version of coding rules was sufficient to improve reliability by the greatest extent. By the fourth coding round, the percentage agreement exceeded 99.0% in all categories except for 'intervertebral disc degeneration' and 'other endplate irregularities'. These results were reinforced by those of the second group of inexperienced clinicians who displayed a mean *inter-rater *percentage agreement of 99.0% and mean *intra-rater *percentage agreement of 98.7%.

Similarly, the levels of reliability (Kappa coefficients) achieved by the fourth coding round for the first group of inexperienced clinicians exceeded 0.85 in all categories other than for 'endplate irregularities', which would all be classified by the Landis criteria as showing excellent reliability [19]. In addition to the lower Kappa value observed for intervertebral disc degeneration in coding round 1, a lower value was also observed for nerve root compromise in coding round 3. Similar to disc degeneration, greater discordance for nerve root compromise and also for endplate irregularities might be explained by a relatively larger number of terms used to describe these findings. These results were also reinforced by those obtained by the second group of inexperienced clinicians. Their *inter-rater *Kappa coefficients all exceeded 0.92, except for 'nerve root compromise' and their *intra-rater *Kappa coefficients all exceeded 0.88, except for 'nerve root compromise' and 'disc bulge'. To our knowledge, no other studies have explored the reproducibility of this task and therefore it is not possible to compare our results with those reported in other literature.

As the level of training required to transform narrative MRI reports to quantitative data was uncertain, this study compared inter-rater reproducibility between a group of trainee clinicians and highly trained MRI coders. The results suggest that trainee clinicians displayed equal reproducibility as experienced researchers in performing this task, when trained with simple coding rules. Collectively, these data suggest that with minimal training and the introduction of basic coding rules, trainee clinicians can accurately and reliably extract pathoanatomic information from MRI narrative reports.

In the absence of radiologists being available to quantify pathoanatomic findings directly from an MRI console, having other personnel quantify these findings from narrative reports is the next best alternative, particularly in the context of workforce limitations and limited budgets for research projects. Our evidence that inexperienced clinicians can perform this task with high reproducibility has implications for the planning, feasibility and cost of research and quality assurance activities. However, these findings are derived within a research/quality assurance context and should not be inferred to have implications for clinical practice. Examination of the reliability of inexperienced clinicians at extracting *clinically relevant *information from MRI reports was not part of the current study.

Another potential alternative is to create software that could screen narrative reports for key phrases, and thereby, automate the process of coding pathoanatomic findings. However, this would need to overcome the challenge of the disparate and inconsistent ways in which radiologists report the same findings and ideally would need to be shown to have at least comparable reproducibility as human raters.

The design of this study has three strengths. First, the electronic coding matrix contained an extensive list of common pathoanatomic observations reported in MRI narrative reports. The matrix also provided an efficient and dependable mechanism to enter and store data. Second, we established reproducibility using a group of trainee clinicians in order to determine whether this task could be performed well by a relatively inexperienced group of people, as this reflects common workforce characteristics in large research projects. We then compared their level of reproducibility to that of highly trained researchers. Thirdly, we replicated the findings in a new group of trainee clinicians that did not participate in establishing the coding rules, thereby extending the generalisability of the results.

These results should, however, be considered in the context of some limitations. The number of MRI reports used in each coding round (n = 20) was chosen arbitrarily and might be considered small. However, depending on the pathologies and vertebral levels involved in each comparison between raters, this resulted in between 120 and 140 observations per rater per round for each pathology, and a total of 1700 observations per rater per round across all the pathologies combined. A post hoc power calculation showed adequate power to detect the Kappa values typically observed. Our decision to compare three trainee clinicians in each group was also arbitrary and based on these people also being part of a larger research programme. Furthermore, the trainees consisted of physiotherapy students only. It is possible that these estimates of reproducibility may vary, depending on the number of raters and their professional discipline. In this study, percentage agreement was calculated based on raters' nominating the presence of a condition by selecting a pathoanatomic category and nominating absence of a condition by not selecting a pathoanatomic category. Given there were 85 potential selections per patient, the low prevalence of some pathoanatomic findings resulted in a large proportion of 'absent findings', which may have inflated inter-rater agreement. However, this approach for calculating percentage agreement is widely reported in the literature [12-14]. Another potential limitation is that the experienced clinicians used for comparison were not radiologists. This was a pragmatic comparison, as it is not common that research radiologists are available for coding tasks in large-scale research projects. Therefore the comparison clinicians were chosen as representative of research personnel more commonly available for this task.

## Conclusions

Results from this study provide evidence of very high inter-rater and intra-rater reproducibility for inexperienced clinicians in extracting pathoanatomic information from MRI narrative reports. These inexperienced clinicians also showed high reproducibility compared with researchers highly trained in this task. Small improvements in reproducibility can be achieved with the introduction of coding rules when transforming narrative information to quantitative data. These findings suggest that for research and quality assurance purposes, the quantification of pathoanatomic findings contained in narrative MRI reports can be performed by inexperienced clinicians.

## Competing interests

The authors declare that they have no competing interests.

## Authors' contributions

All authors participated in the design of the study, its coordination, its statistical analysis, and read and approved the final manuscript. PK conceived of the study and designed the database. AMB and PK drafted the manuscript.

## Supplementary Material

Additional file 1 The final set of decision rules used to extract pathoanatomic findings from MRI narrative reports. Click here for file
