# Supplementary material for: Inexperienced clinicians can extract pathoanatomic information from MRI narrative reports with high reproducibility for use in research/quality assurance
Source: Chiropr Man Therap. 2011 Jul 21;19:16. doi: 10.1186/2045-709X-19-16 (PMC3154850; doi:10.1186/2045-709X-19-16)
Supplement: Additional file 1 — The final set of decision rules used to extract pathoanatomic findings from MRI narrative reports. [file 2045-709X-19-16-S1.PDF]

Additional file 1 - The final set of decision rules used to extract pathoanatomic findings from MRI narrative reports

Pathology	Expressions used in the narrative reports
Disc degeneration	- Age appropriate degeneration<br>- Reduced water content<br>- Reduced disc height<br>- Light/moderate/severe degeneration<br>- Collapsed intervertebral space<br>- Spondylosis
Disc herniation	- Small/medium/large herniation<br>- Sequestration<br>- Prolapse<br>- Prolapse-like protrusion*<br>- Prolapse/protrusion*
Disc bulge	- Small/medium/large<br>- Prolapse-like protrusion*<br>- Prolapse/protrusion*
Nerve root compromise	- Compression of nerve root<br>- Affected nerve root<br>- Dislocation of nerve root<br>Nerve root at the same disc level (nerve root for lower level coded at the disc level below)<br>- NO CODING of nerve root compromise when the following expressions are used: ”touching …”, “contact…”, “enhancement of …”
Modic changes	- For “endplate changes”: ONLY if types 1 or 2 are mentioned<br>- Terminal plate changes<br>- Fatty degeneration in the endplate or terminal plate are coded as Modic type 2
Spondylolisthesis (antero/retro), spondylolysis	- Light/small<br>- Moderate/medium<br>- Large/severe
Stenosis	- Foraminal stenosis<br>- Central stenosis<br>- Antero-posterior diameter below 10 mm.
Scoliosis	- Scoliosis with an apex is coded at that level<br>- Scoliosis without an apex is coded for all levels (Th12-L5). Note: “lumbar scolioses” are not coded for Th12.
Osteophytes	- Bony spurs<br>- Traction spurs<br>- Spondylosis
Facet joint arthrosis	- Attrition of facet joint<br>- Degeneration of facet joint
Other endplate changes	- Scheurmann sequelae<br>- Schmorls nodes (herniations in the endplate are coded as Schmorls nodes)<br>- Endplate defects<br>- Findings in the endplate/terminal plate<br>- Endplate changes if types are NOT indicated

* The evaluation of disc herniations and disc bulges can be confusing in Danish narrative reports because the International nomenclature[1] and the Danish nomenclature for disc herniations are contradictory, but both are used.. For example, “bulge” (protrusion of disc tissue of more than 50% of the circumference[1]) in the International nomenclature is “protrusion” in the Danish nomenclature. This is confusing because “protrusion” means less than 50% of the circumference[1] in the International nomenclature, and this is called “herniation or prolapse” in the Danish nomenclature. Because of this, the coding of disc herniations and disc bulges in this study was subject to interpretation from the clinicians.

- ONLY MRI-scans are coded, not radiographs
- Th12 is NOT included as lumbar level
- “Upper lumbar levels” = 3 upper levels (L1-L3)
- “Lower lumbar levels”= 3 lowest levels (L4-S1)
- A positive finding is defined as = ”a hint of…”, ”possible…”, ”potential…”, “relative…”, “affected… (except for stenosis)”

1. Fardon DF, Milette PC. Nomenclature and classification of lumbar disc pathology. Recommendations of the Combined Task Forces of the North American Spine Society, American Society of Spine Radiology, and American Society of Neuroradiology. *Spine* 2001;26:E93-E113.
